# Genome sequences of three methicillin-resistant *Staphylococcus aureus* strains isolated from subclinical mastitic cows

**DOI:** 10.1128/mra.00397-25

**Published:** 2025-07-23

**Authors:** Eaftekhar Ahmed Rana, Sifatun Nur, Md Abul Fazal, Mahabub Alam, A. S. M. Lutful Ahasan, Md Jahedul Hasan Rocky, Tofazzal Md Rakib

**Affiliations:** 1 Department of Microbiology and Veterinary Public Health, Faculty of Veterinary Medicine, Chattogram Veterinary and Animal Sciences University 130057, Chattogram, Bangladesh; 2 Department of Aquaculture, Faculty of Fisheries, Chattogram Veterinary and Animal Sciences University 130057, Chattogram, Bangladesh; 3 Department of Animal Science and Nutrition, Faculty of Veterinary Medicine, Chattogram Veterinary and Animal Sciences University 130057, Chattogram, Bangladesh; 4 Department of Anatomy and Histology, Faculty of Veterinary Medicine, Chattogram Veterinary and Animal Sciences University 130057, Chattogram, Bangladesh; 5 Department of Medicine and Surgery, Faculty of Veterinary Medicine, Chattogram Veterinary and Animal Sciences University 130057, Chattogram, Bangladesh; 6 Department of Pathology and Parasitology, Faculty of Veterinary Medicine, Chattogram Veterinary and Animal Sciences University 130057, Chattogram, Bangladesh; University of Maryland School of Medicine, Baltimore, Maryland, USA

**Keywords:** *Staphylococcus aureus*, MRSA, multidrug resistance, mastitic cow, antimicrobial resistance

## Abstract

Methicillin-resistant *Staphylococcus aureus* (MRSA) in bovine milk poses a significant zoonotic and antimicrobial resistance threat. This study identifies three MRSA strains from subclinical mastitic (SCM) cow milk, carrying resistance genes to β-lactams, lincosamides, macrolides, streptogramin B, and aminoglycosides, underscoring the urgent need for improved hygiene and surveillance in dairy production.

## ANNOUNCEMENT

Methicillin-resistant *Staphylococcus aureus* (MRSA) in bovine milk poses a significant threat to both animal and public health due to its antimicrobial resistance and zoonotic potential (1). Its presence in raw and pasteurized milk increases the risk of clinical management and human transmission, especially among those in close contact with dairy animals or consuming unpasteurized products (2, 3). Concerns underscore the urgent need for improved hygiene practices and robust surveillance in the dairy industry to mitigate MRSA spread. Three subclinical mastitis (SCM) milk samples were collected from the Cumilla district, Bangladesh, and 20 µL milk was inoculated on 5% bovine blood agar followed by subculture of characteristic colony on Mannitol Salt Agar with incubation at 37°C for 24 h. *Staphylococcus aureus* was confirmed by subsequent Gram staining, biochemical, and *nuc* gene-specific PCR (4). Antimicrobial sensitivity profiling using 12 antimicrobials representing different classes resulted in multidrug resistance based on CLSI guidelines (5). Genomic DNA was extracted from two to three isolated subcultured colonies on 5% bovine Blood Agar using an AllPrep Bacterial DNA/RNA/Protein Kit (Qiagen, Hilden, Germany), and purity was assessed with a Nanodrop One (Thermo Fisher Scientific, MA, USA). DNA libraries were prepared using the Nextera XT DNA Library Preparation Kit (Illumina, CA, USA). Genome sequencing was conducted on an Illumina NextSeq2000 platform generating 2 × 150 bp paired-end reads. Raw data quality, including read quality, GC content, and adapter contamination, was assessed using FastQC v0.12.1 (6). Trim Galore v0.6.5dev (7) was used to trim low-quality bases (Q < 20) and adapter sequences, followed by normalization of read coverage with bbnorm v39.08 (8) to reduce redundancy. *De novo* genome assembly was performed using Unicycler v0.4.8 (9) with subsequent polishing using Pilon v1.24 (10) to enhance accuracy. Genome assembly quality was assessed using QUAST v5.2.0 (11), while Samtools v1.13 (using htslib 1.13 + ds) (12) was used for alignment file manipulation and indexing. The *de novo* genome assembly conducted through the BV-BRC (13) comprising the abovementioned tools yielded coverage ranging from 146× to 339×, with raw reads between 3,035,549 and 6,710,480. The assembled genomes were annotated using the National Center for Biotechnology Information Prokaryotic Genome Annotation Pipeline (PGAP) v6.9 (14) and PATRIC (15) with the in-built RASTtk pipeline to identify the species, *Staphylococcus aureus*. Transport proteins were identified via TCDB (16), drug targets using DrugBank v6.0 (17), and additional analyses performed for CRISPR arrays and prophages using CRISPR CasFinder (18), plasmids using PlasmidFinder 2.1 (19), and integrative and conjugative elements using ICEFinder (20). All software parameters were set to default, unless specified otherwise. Detailed genomic characteristics are provided in Table 1.

**TABLE 1:** Genomic features of the sequenced three *Staphylococcus aureus* strains isolated from subclinical mastitic cows in Cumilla, Bangladesh^*a*^

Feature	Value for strain:		
	BioB-2	BioB-3	BioB-4
Species	*Staphylococcus aureus*	*Staphylococcus aureus*	*Staphylococcus aureus*
GPS coordinate of sampling sites	23.481061 N 91.069869 E	23.522098 N 90.782914 E	23.44875 N 91.079426 E
Antibiogram result (disk diffusion method)	R: AMP, FOX, OX, FEP, E, CN, SXT, TE S: AMC, CRO, IMP, CIP	R: AMP, AMC, CRO, FOX, OX, FEP, E, CN, TE S: IMP, CIP, SXT	R: AMP, AMC, CRO, FOX, OX, FEP, E, TE S: IMP, CIP, CN, SXT
BioProject ID	PRJNA1190104	PRJNA1190104	PRJNA1190104
BioSample accession number	SAMN45025667	SAMN45025668	SAMN45025669
SRA accession number	SRS23333889	SRS23333890	SRS23333891
Genome assembly accession number	GCA_047604675.1	GCA_047604515.1	GCA_047604495.1
GenBank accession number	JBJLRB000000000	JBJLRA000000000	JBJLQZ000000000
FastQC Phred score	34	34	34
Raw reads	6,710,480	4,498,755	3,035,549
Coverage	339x	219x	146x
Contig count	31	24	41
Coarse consistency (%)	100	100	100
Fine consistency (%)	99.7	99.7	99.7
Completeness (%)	100	100	100
Contamination (%)	0	0	0
Genome size (bp)	2,761,071	2,782,581	2,797,289
Contigs N50 (bp)	219,219	329,157	137,483
Contigs L50	5	4	5
Largest contig (bp)	402,935	536,524	380,235
Guanine–cytosine content (%)	32.75696	32.72947	32.637028
Genes (total), PGAP	2,763	2,791	2,799
CDS (PGAP; PATRIC)	2,641; 2,606	2,668; 2,624	2,634; 2670
tRNA	55	55	57
rRNA	4	4	4
CDS ratio	0.9438366	0.9430094	0.9544956
Hypothetical CDS	510	507	568
Hypothetical CDS ratio	0.2943208	0.29115853	0.31722847
PLFAM CDS	2,540	2,557	0
PLFAM CDS ratio	0.9746738	0.97446644	NA
Hypothetical proteins	513	507	570
Proteins with functional assignments	2,093	2,115	2,100
Proteins with EC number assignments	735	737	740
Proteins with GO assignments	607	609	613
Proteins with pathway assignments	532	533	536
Proteins with subsystem assignments	975	973	974
Proteins with PATRIC genus-specific family (PLfam) assignments	2,540	2,557	0
Proteins with PATRIC cross-genus family (PGfam) assignments	2,573	2,593	2,629
Proteins with FIGfam assignments	0	0	0
Transporter (TCDB)	98	99	100
Drug target (DrugBank)	33	36	34
Antibiotic resistance genes (PATRIC)	39	40	40
ResFinder 4.6.0 (acquired; chromosomal)	*str, mecA; 23S, dfrB, fusA, grlA, grlB, gyrA, ileS, pbp2, pbp4, rpoB, pbp4* promoter	*mecA, blaZ, erm(C); 23S, dfrB, fusA, grlA, grlB, gyrA, ileS, pbp2, pbp4, rpoB, pbp4* promoter	*mecA, blaZ; 23S, dfrB, fusA, grlA, grlB, gyrA, ileS, pbp2, pbp4, rpoB, pbp4* promoter
AMR class (based on ResFinder 4.6.0 output)	β-lactam	β-lactam, lincosamide, macrolide, streptogramin b	β-lactam, aminoglycoside
Plasmid finder 2.1	rep7a (contig23: 2056..2895)	rep5a (contig13: 16621..17481), rep16 (contig13: 18772..19515), rep10 (contig18: 1881..2356)	repUS5 (contig21: 33881..35455), rep21 (contig21: 4961..5956)
Integrative and conjugative elements (ICEs)	1(2,745,519..2,754,895, 9,377 bp)	1(2,111,899..2,114,925, 3,027 bp)	2 (2,759,741..2,794,168, 34,428 bp; 1,058,307..1,163,904, 105,598 bp)
No. of clustered interspaced short repeats (CRISPR) (number of cas clusters) (name of cas genes)	4(1) (cas4_Type IA)	6(1) (cas4_Type IA)	8(1) (cas3_TypeI)

^*a*^ AMP = ampicillin, AMC = amoxycillin/clavulanic acid, CRO = ceftriaxone, FOX = cefoxitin, OX= Oxacillin, FEP = cefepime, IPM = imipenem, CIP = ciprofloxacin, E = erythromycin, CN = gentamicin, SXT = trimethoprim/sulfamethoxazole, and TE = tetracycline. R = resistant, S = sensitive, and NA = not applicable.

Acquired and chromosomal resistance genes were identified in all three MRSA strains using ResFinder 4.6.0 (21). Methicillin-resistant *mec*A gene and resistance to β-lactam antibiotics were present in all three strains. Additionally, resistance to lincosamide, macrolide, streptogramin B, and aminoglycoside was found in BioB-3 and BioB-4 strains (Table 1). This study highlights the need for effective surveillance and control measures to prevent the spread of MRSA within dairy herds and to humans.

## Data Availability

The accession numbers for the genomes of the three *Staphylococcus aureus* strains are shown in Table 1. All genomes are available under BioProject accession number PRJNA1190104.
